# Supplementary material for: Effects of ε-Poly-L-Lysine Combined with Wuyiencin as a Bio-Fungicide against Botryris cinerea
Source: Microorganisms. 2022 May 5;10(5):971. doi: 10.3390/microorganisms10050971 (PMC9146948; doi:10.3390/microorganisms10050971)
Supplement: Supplementary file 1 [file microorganisms-10-00971-s001.zip › microorganisms-1703147-supplementary.pdf]

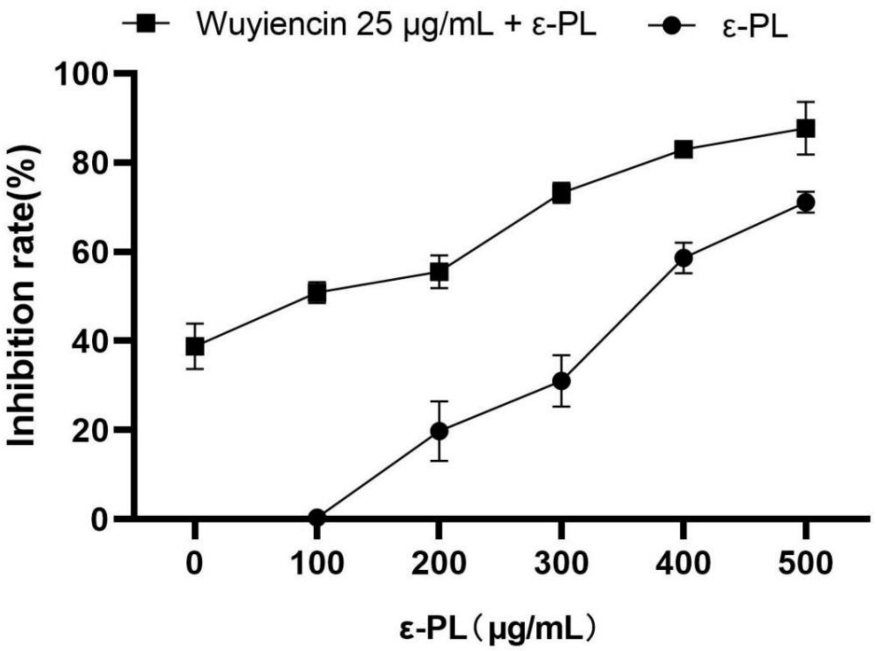

Figure S1. Inhibitory effect of various concentrations of ε-PL and wuyiencin on the mycelial growth of *Botrytis cinerea.*

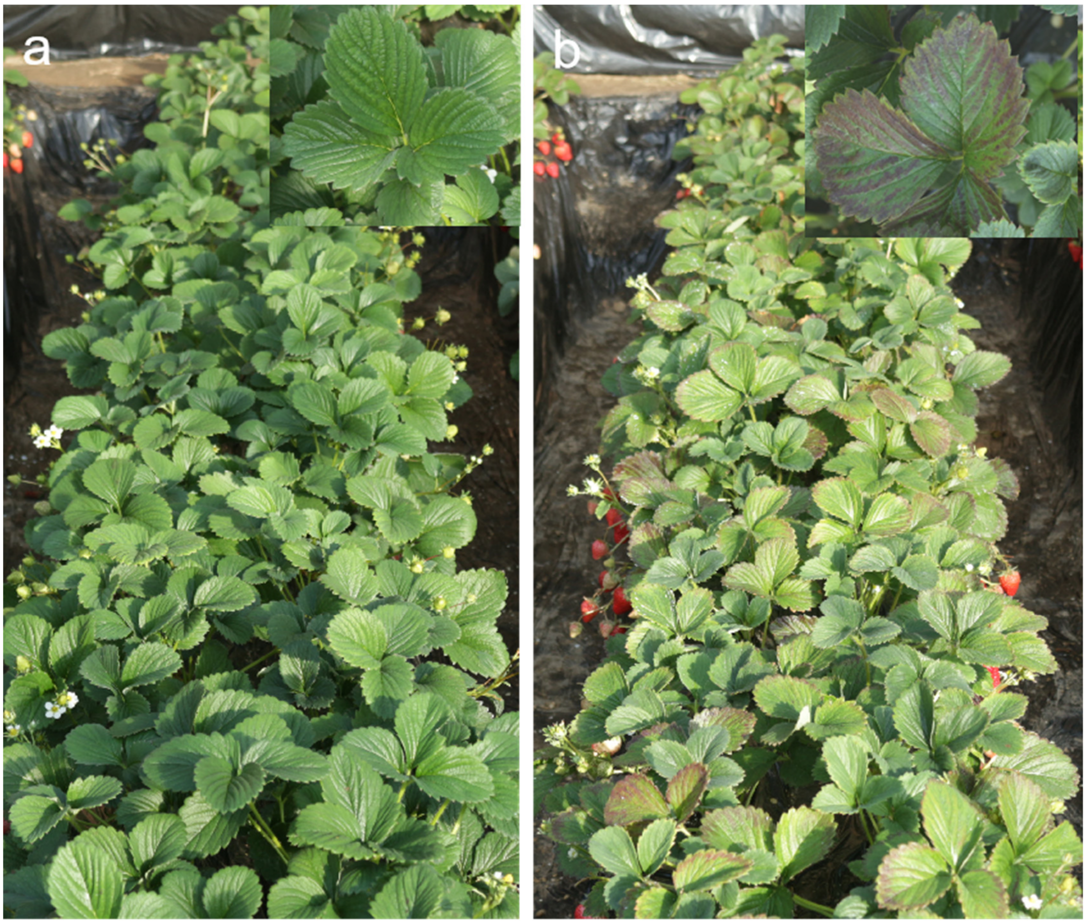

Figure S2. Effects of ε-PL and wuyiencin on strawberry leaves.

(a): Treatment with 2000 μg/mL ε-PL+ 60 μg/mL wuyiencin; (b): Treatment with 2000 μg/mL ε-PL.
